# Gold Nanorod Density-Dependent Label-Free Bacteria Sensing on a Flake-like 3D Graphene-Based Device by SERS

**DOI:** 10.3390/bios13110962

**Published:** 2023-10-30

**Authors:** Md Imran Hossain, Sitansu Sekhar Nanda, Sooheon Cho, Bom Lee, Bum Jun Kim, Jae-Young Choi, Dong Kee Yi

**Affiliations:** 1 Department of Chemistry, Myongji University, Yongin 17058, Republic of Korea; imranhossain@mju.ac.kr (M.I.H.);; 2 School of Advanced Materials Science and Engineering, Sungkyunkwan University, Suwon 16419, Republic of Korea; 3 SKKU Advanced Institute of Nanotechnology (SAINT), Sungkyunkwan University, Suwon 16419, Republic of Korea

**Keywords:** AuNRs, SERS, Raman spectroscopy, bacteria sensing, graphene, detection

## Abstract

Surface-enhanced Raman spectroscopy (SERS) is an effective technique for biosensing, enabling label-free detection of biomolecules with enhanced sensitivity. There is a tremendous probability of signal failure in Raman frequencies because of the scattering of the Raman radiation in liquids, effective SERS improvement is required to reduce this issue when considering liquid specimens. We examined a liquid bacterial sample, investigating the electrostatic interactions of the bacterial samples with gold nanorods (AuNRs) and graphene. We established a voltage-gated 3D graphene functionalized with an AuNR-based device on the silicon substrate for SERS measurements when the applied voltage ranges from 0 to 3 V. Moreover, AuNRs density-susceptible bacterial sample analysis with varied concentrations of bacterial samples has also been described. Using bacterial SERS analysis, the bacterial components amide II (1555–1565 cm^−1^) and amide III (1250–1350 cm^−1^) have been discovered for both bacteria, Gram-positive, *Listeria monocytogenes* and Gram-negative, *Salmonella typhi*. Our fabricated device affords an interesting label-free, rapid, and reproducible bacterial sample analysis based on the density of the AuNRs when functionalizing flake-like 3D graphene, which can help facilitate label-free bacteria sensing platforms.

## 1. Introduction

Graphene is a two-dimensional atomically thick substance that has been enormously researched for extensive applications because of its remarkable electrical and thermal properties [1,2,3,4,5]. The vast number of π electrons make active charge transfer with biomolecules possible in graphene. This study focused on the chemical mechanism of graphene using a method called surface-enhanced Raman spectroscopy (SERS). Because of the limitations of SERS performance on the enhancement factor (less than 100) [6,7,8], metal nanoparticles have been introduced to the surface of graphene [9,10]. Researchers use metal nanoparticles to achieve higher enhancement through the electromagnetic mechanism. To completely optimize the unified effects of the two CM [11] and EM [12] enhancements, several graphene-metal-based hybrid structures have been developed. Functionalization of graphene via various approaches such as metal nanoparticles (NPs) and graphene hybrid materials has been introduced previously [13,14]. Flake-like crumpled three-dimensional (3D) graphene produces an amplified surface area which presents opportunities for optical sensing applications [15,16,17,18,19,20,21]. Graphene utilizes multiple roles as a flat surface [22], an excellent absorber [23], and prevents oxidation of metal nanostructures [24]. The 3D flake-like structure amplifies the surface area and volumetric density of metal nanoparticles, leading to a significant enhancement in Raman intensity. This allows the utilization of the entire laser focal volume in three dimensions. We have applied an electrostatic field across the silicon substrate, ranging from 0 to 3 V, that can control the fermi level of graphene and the resonance frequency can also be controlled while biomolecule sensing [25]. By controlling the electronic properties of graphene, dissimilar bacteria provide dissimilar vibrational properties on the graphene because the surface charge on the bacterial membrane can be measured even without Raman spectral analysis [26,27,28,29]. There are many difficulties with liquid Raman measurements. Signal loss results from scattering from an inconsistent bacterial suspension with a varying dielectric constant. With drying, the bacterial suspension presumably contributes insufficient interrogation time for bacterial identification. To overcome this issue, we have introduced wafer-scale grown 3D graphene-AuNR hybrid systems for bacterial detection. The highest SERS intensity is attained when the polarization of the excitation laser aligns with the longitudinal surface plasmon mode of nanoparticles (NPs). AuNRs can amplify the Raman signal of graphene with an enhancement factor of up to ~1000 fold [30]. Approaches for label-free biosensing using vibrational spectroscopy promise fast and accurate detection of biological samples without significant cell damage [31,32]. However, the bacterial suspension in the environment during the measurement has a high risk of exposure to the individual and it dries over some time. So, there are some limitations to analyzing several samples. Most of the clinical samples are available in a liquid form and our approach is to analyze the bacterial suspension in a rapid and label-free manner. Based on our previous study on SERS for bacterial fingerprint analysis, we have analyzed the enhancement of the graphene-mediated system by the charge transfer between bacteria and graphene using voltage-gated graphene [33]. However, EM enhancement together with CM is still unresolved for the label-free bacterial sample analysis by SERS, which may explain the significant large-area SERS enhancement.

There are several papers that have shown the improvement in SERS enhancement, introducing several techniques such as the combination of metal–organic frameworks (MOFs) with plasmonic metal nanoparticles [34], silver nanoparticles with porous silicon substrate [35], metal-modified montmorillonite [36], AuNRs functionalized zirconium-based MOFs [37], and etched-spiky Au@Ag plasmonic superstructures [38]. Our system can analyze bacteria samples without labels using a reusable biosensing device made of graphene and AuNRs.

**Figure 1:**
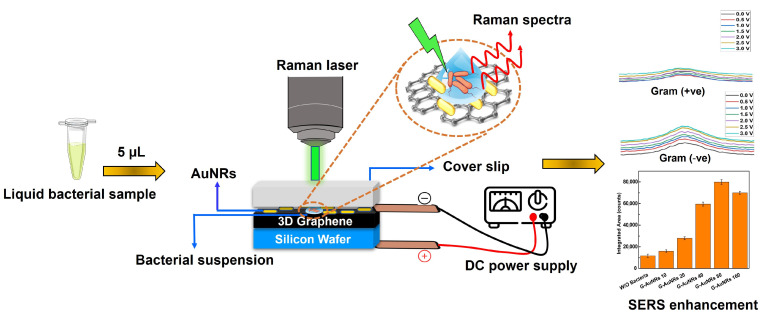
Schematic illustration of the 3D graphene-AuNR-mediated device as a label-free bacteria sensing platform and analyzing the integrated area in SERS enhancement with the different densities of AuNRs on the device.

Herein, we show a SERS platform for liquid bacterial sample analysis with large-area SERS enhancement by utilizing both CM and EM. Particularly, we demonstrate the surface charge effects of bacteria by the interaction between the different densities of AuNRs and 3D graphene to determine the area enhancement by SERS calculating the area under the peak values and the different concentrations of bacteria for SERS analysis. We use Gram-negative *S. typhi* and Gram-positive *Listeria monocytogenes* as model bacteria for studying the interaction of bacteria between the AuNRs and 3D graphene in a 10 × 10 mm fabricated device when the applied voltage ranges from 0 to 3 V. In this study, our Raman spectra results showed that the spectral values are consistent with their corresponding bacteria and are reproducible. The interaction between the graphene and bacterial surface is known and the chemical potential of the bacterial membrane can change the vibrational properties of graphene [39]. In label-free biosensing, sample preparation is often not required for label-free biosensors due to their inherent ability to directly interact with complex biological samples. Label-free biosensors are designed to detect and quantify biomolecules or analytes based on their intrinsic properties, such as mass, charge, refractive index, or electrical conductivity. This eliminates the need for tagging or labeling the target molecules with fluorescent or other markers. We detect the surface charge of the bacteria for both Gram-positive and Gram-negative bacteria. We showed a schematic view of our research comprising the Raman setup and device illustration in Figure 1.

## 2. Materials and Methods

### 2.1. 3D Flake-like Graphene and Hydrophobic AuNR Synthesis

The metal–organic chemical vapor deposition (MOCVD) method is used to synthesize flake-like 3D graphene directly on a SiO_2_/Si substrate using a modified horizontal MOCVD reactor system and supply metal precursor (bis(t-butyl acetoacetate) copper(II), 99%, STREM Chemicals Inc., Boston, MA, USA) which is clearly described in our previous publication [40]. Briefly, the MOCVD system comprises three main components: gas supply lines for the reaction, a bubbler/heater containing the organometallic precursor bis(t-butyl acetoacetate) Cu(II) to provide the copper catalyst, and a horizontal CVD reaction chamber made of glass tubing. At first, argon gas was purged three times to remove air molecules, the entire temperature was raised to 1050 °C for 1 h, and the copper precursor maintained a temperature of 100 °C to initiate the sublimation process as a precursor. The copper precursor evaporated and introduced to the argon gas through the bubbler. Thereby the nanostructured graphene was deposited on the silicon substrate by flowing CH_4_, H_2_, and copper-containing Ar, respectively.

CTAB (cetyltrimrthyalammonium bromide)-stabilized AuNRs in an aqueous solution were prepared using our previous publication [41]. Then, the excess CTAB in DI water was removed by centrifugation. The AuNRs were redispersed into an aqueous phase and chloroform containing 10 mM dodecanethiol by a 1:1 ratio. The two-phase system was then vigorously stirred. During stirring for 24 h, the color of the aqueous phase was transferred to the organic phase, washed, and redispersed into chloroform.

### 2.2. Bacteria Culture

*Salmonella typhi* [KCCM 40253] and *Listeria monocytogenes* [ATCC 15313] were cultured in Luria–Bertani broth (Difco, BD, Franklin Lakes, NJ, USA) medium until the optical density came to 0.15, 0.10, and 0.05 for three different bacterial suspensions. Afterward, the LB medium was removed by subjecting it to centrifugation at 8000 rpm for 10 min, removing the supernatant, and dispersed in DI water for 3 different concentrations of bacteria. The concentration of the bacteria was calculated by the previously introduced method [42]. The three different concentrations of the bacteria are 1.2 × 10^8^, 8.0 × 10^7^, and 4.0 × 10^7^ cells/mL.

### 2.3. Device Fabrication and Raman Measurements

Our device consists of directly grown MOCVD 3D graphene on a SiO_2_/Si substrate. The electrode was prepared by keeping the device on the hot plate and heating it to 160 °C and some indium was placed on the device edge which covered the area of graphene and we waited 1 min to melt the indium. Thereby, the copper tape was attached to the melted indium and kept at room temperature to harden the attachment between the copper tape and the device. Then, the 4 different concentrations of hydrophobic AuNRs such as 10, 20, 40, and 80 µg/mL with a volume of 20 µL were deposited on the surface (10 × 10 mm) of the graphene by solvent evaporation.

Raman spectroscopy was performed with a commercial Raman system (WITec, UHTS-300 spectrometer, Ulm, Germany), equipped with a YAG laser, wavelength of 532 nm, a laser spot size of 1 μm and a power of 70 μW was used during the measurement. For collecting the Raman spectra, a distance of 8 mm, and a 10× objective lens from Olympus were used and the acquisition time was 5–10 s. For every measurement, 5 µL of the bacterial suspension was placed into the well of the device. We have performed all the measurements three times to maintain the accuracy associated with the integrated area enhancement.

### 2.4. Characterization of the 3D Flake-like Graphene and Hydrophobic AuNRs

The characterization of flake-like 3D graphene has been performed by using a scanning electron microscope (SEM), and the Raman spectra of graphene (see Figure S2A). The scanning electron microscope (SEM) image revealed the presence of a flake-like, rough edge on the graphene substrate (as depicted in Figure S2B). Furthermore, the Raman spectra obtained from this three-dimensional flake-like graphene exhibited the characteristic Raman peaks associated with graphene. Specifically, these peaks included the D peak at ~1370 cm^−1^, the G peak at ~1585 cm^−1^, and the 2D peak at ~2700 cm^−1^. The copper precursor acts as a catalyst to enhance the formation of graphene, just like regular Cu catalytic substrates. Therefore, the precursor remains in gas form and does not deposit on the substrate. We also did not observe any Cu-related peak on Raman spectra above baseline which normally forms in the 300~700 regime.

Since the described synthesis method is a CVD-based method, the size of graphene is continuous throughout the entire substrate, which is ~1 cm^2^. the size of the flake-like protruded part of graphene film varies, but typically does not exceed 100 nm.

The characterization of the AuNRs was achieved by analyzing the UV–vis spectrophotometer (Scinco Co., Ltd., Seoul, Republic of Korea) with a quartz cell in the wavelength range 450–850 nm. However, the hydrophobication of the AuNRs was characterized by FT-IR manufactured by Thermo Scientific, Waltham, MA, USA. The FTIR spectra at 2550–2600 cm^−1^ represent S-H stretching that confirmed the thiol functional group in AuNRs, and the visual observations of the AuNRs from aqueous to organic solvent also confirm the hydrophobication of the AuNRs and redispersion in an organic solvent (see Figure S1). The measured spectra showed the extinction resonance peaks at ~520 nm and localized surface plasmon resonance at ~677 nm. The shape of the materials was confirmed by a high-resolution transmission electron microscope (JEM2100F, JEOL, LTD, Pleasanton, CA, USA). The rod-like AuNRs were characterized and the average aspect ratio of the AuNRs is 2.45 (see Figure S2C,D). A previous study showed that smaller sizes result in a stronger SERS signal due to increased lightning rod effect and reduced radiation damping [43].

Atomic force microscopy (AFM) is one of the most acceptable techniques for measuring the height profile of the adsorbed molecules on a substrate. The height profiles of the adsorbed and unabsorbed AuNRs on the graphene surface have been compared in Figure S3. Figure S3A represents the height profile and the image of the flake-like 3D graphene. Conversely, Figure S3B–E depicts the different concentrations of AuNR deposition on the flake-like 3D graphene surface from low to high concentrations, respectively.

## 3. Results and Discussion

### 3.1. Measurement of Bacterial SERS Enhancement on Different Densities of AuNRs

Herein, we introduced a 3D graphene-AuNR-based voltage-gated device for liquid bacterial SERS analysis for large-area SERS enhancement. We study how the density of AuNRs on 3D graphene affects bacterial SERS analysis. We also study how bacterial surface charge affects the analysis of bacterial SERS using a 3D graphene system. We analyzed the best amount of AuNRs on 3D graphene to improve SERS spectra in both CM and EM enhancements. As a result, we successfully detected the liquid bacterial SERS signature even at a low concentration of 4.0 × 10^7^ cells/mL. Moreover, we are the first to report the functionalization of 3D graphene surfaces with the AuNRs and it enables both CM and EM ways together to study concentration-based bacteria sensing in a label-free biosensing platform. However, electromagnetic enhancement for graphene has been widely studied [44,45]. EM occurs due to the local electromagnetic field associated with the localized surface plasmon resonance (LSPR) of the AuNRs. The plasmonic properties of the nanostructured AuNRs enhance the optical absorption rate in graphene. Additionally, CM enhancement has been studied in many previously published articles [46,47]. The CM enhancement mechanisms rely on the charge-transfer enhancement originating from the bacterial interaction with the 3D graphene-AuNR-mediated system. The charge transfer enhancement can be further investigated by density functional theory.

**Figure 2:**
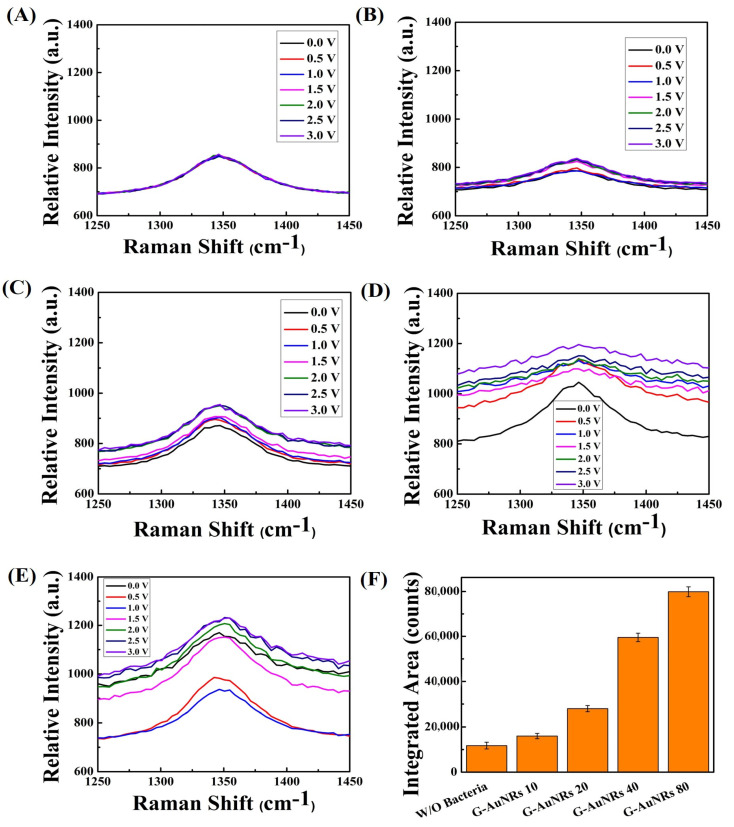
(**A**) D band (~1370 cm^−1^) of graphene with crumpled 3D graphene-AuNRs (80 µg/mL, 20 µL, 10 × 10 mm of graphene) without bacteria (DI water). (**B**–**E**) D band of the voltage-gated crumpled 3D graphene-AuNRs (10, 20, 40, and 80 µg/mL), a voltage applied from 0 to 3 V. (**F**) Area under the peak values for the different density of AuNRs deposited on crumpled 3D graphene for D band of graphene with the Gram-positive *Listeria monocytogenes* bacteria when a voltage applied of 3 V.

**Figure 3:**
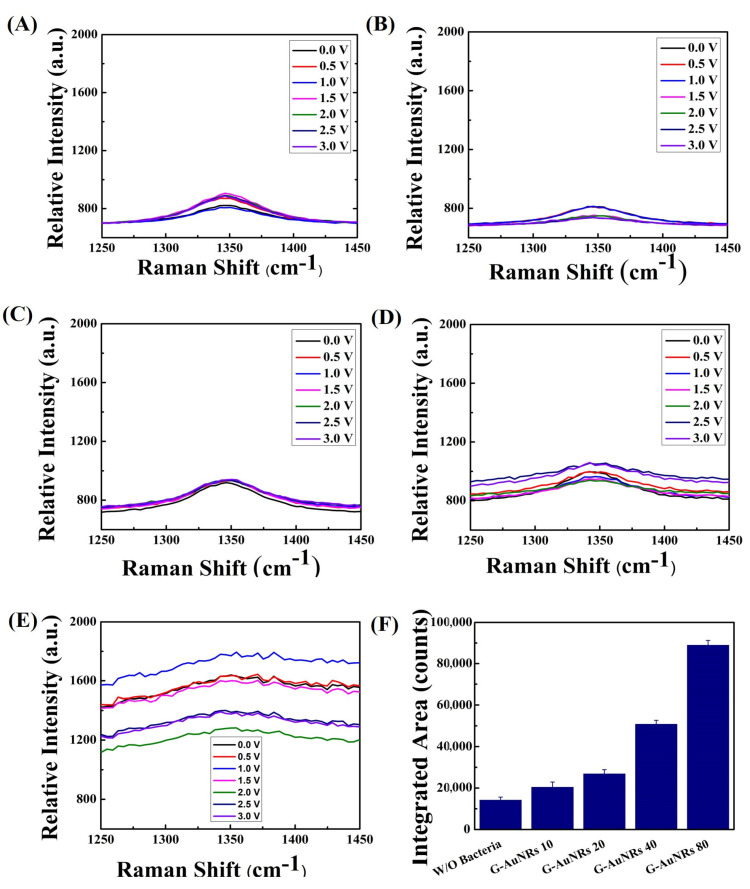
(**A**) The D band (~1370 cm^−1^) of crumpled 3D graphene-AuNRs with graphene alone (AuNRs, 80 µg/mL, 20 µL, 10 × 10 mm of graphene) in the absence of bacteria (DI water). (**B**–**E**) The D band of voltage-induced crumpled 3D graphene-AuNRs at different concentrations (10, 20, 40, and 80 µg/mL), with a voltage range of 0 to 3 V applied. (**F**) The integrated area representing SERS enhancement for varying densities of AuNRs deposited on crumpled 3D graphene with the, specifically for the D band of graphene interacting with Gram-negative *S. typhi* bacteria, under a 3 V applied voltage.

Our synthesized Au rods are sensitive to polarization (direction of the electric field oscillation of the incident light). Figure S3. The UV–vis spectra of AuNRs exhibit certain properties. At a wavelength of approximately 530 nm, the rod absorbs light that corresponds to its transverse oscillation. Similarly, at a wavelength of 677 nm, the rod absorbs light that corresponds to its longitudinal oscillation. These results are in good agreement with the previous report on the optical properties of Au rods [48]. No significant enhancement was found without bacteria (see Figure 2A and Figure 3A). The highest-integrated area for SERS enhancement was found with a high density of AuNR deposition on the graphene surface for D band for Gram-negative bacteria with increasing density of AuNRs (see Figure 3B–E). A related phenomenon was observed for G band also. The Raman intensity is proportional to the incident electromagnetic field to the power of 2. Therefore, if the polarized light hits a well-matched axis of the Au nanomaterials, the intensity will be enhanced, but if the polarized light is not well matched to the axis of the Au nanomaterials, the interaction will not show a clear Raman intensity. According to Smalyukh et al. star-shaped Au particles do not show polarization dependence, unlike Au rods [49]. Therefore, if we use the polarization-controlled light source, we can tune the absorbance and the resulting Raman intensity can also be tuned. In our current system, we have not used polarization-controlled light sources for Raman measurement. As raised by the reviewer, we could perform further studies using a polarization-controlled light system to develop a novel Raman biosensor system.

We investigate the density controllability of the decorated AuNRs on the graphene for bacteria sensors using the SERS platform. We control the density of the AuNRs on the top of the graphene by changing the concentration of the AuNRs during the deposition of AuNRs on the graphene by solvent evaporation, AFM image is shown in Figure S3. Electron charge density increases with the extremely electronegative elements. Through oxygen, graphene’s charge is transferred, as it has the highest electronegativity compared to the other molecules in the amide group. The charge transfer to/from graphene can change graphene’s vibrational properties observed in this experiment by SERS analysis. We have also studied the higher density of AuNRs (160 µg/mL; 20 µL; 10 × 10 mm) deposited on the graphene surface to observe the SERS enhancement profile of D and G band of graphene, respectively (see Figures S4 and S5). Interestingly, we have detected that the deposition of high concentrations of the AuNRs (160 µg/mL) can reduce the SERS enhancement profile and area under the peak values (see Figure S6) for analyzing the bacterial sample due to the hybridization of the AuNRs with graphene thereby the distortion of the graphene lattice that has been previously reported [50]. The most likely mechanism leading to the suppression of the 2D peak in graphene is the hybridization of the d orbitals of Au and the 2p orbitals of graphene [51].

### 3.2. Mechanisms of SERS Enhancement with Bacteria and the Differentiation of Bacteria

The amide group is a constituent of bacteria. We have shown the integrated area for the D band of 3D graphene with increasing density of AuNRs on its surface and the amide III vibrations (1250~1350 cm^−1^) [52] superimposed with the 3D graphene D band hence the increases in the integrated area of the graphene D band for Gram-positive *Listeria monocytogenes*, shown in Figure 2F. Similarly, no significant changes when treated without bacteria using DI water, (AuNRs density 80 µg/mL, 20 µL into 10 × 10 mm graphene) shown in Figure 3A, and the amide III vibrations of bacteria superimposed with the graphene D band with the increasing density of the AuNRs to the 3D graphene surface thereby increasing Raman intensity of D band of graphene when treated with Gram-negative *S. typhi* bacteria. The integrated area enhancement of the D band is shown in Figure 3F.

**Figure 4:**
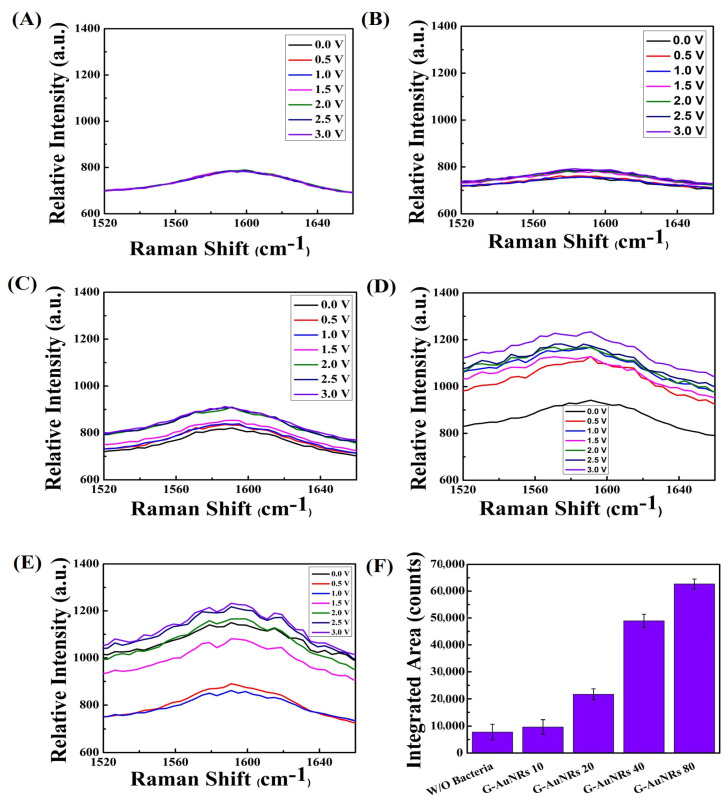
(**A**) The G band of crumpled 3D graphene without bacteria (DI water) displayed G band peaks at 1585 cm^−1^ where the density of AuNRs is 80 µg/mL, 20 µL, 10 × 10 mm of graphene. (**B**–**E**) The G band of voltage-gated crumpled 3D graphene-AuNRs at varying concentrations (10, 20, 40, and 80 µg/mL), subjected to a voltage range of 0 to 3 V. (**F**) The area under the peak values, representing the different AuNRs densities placed on crumpled 3D graphene, for the G band of graphene interacting with Gram-positive *Listeria monocytogenes* bacteria, with a 3 V applied voltage.

**Figure 5:**
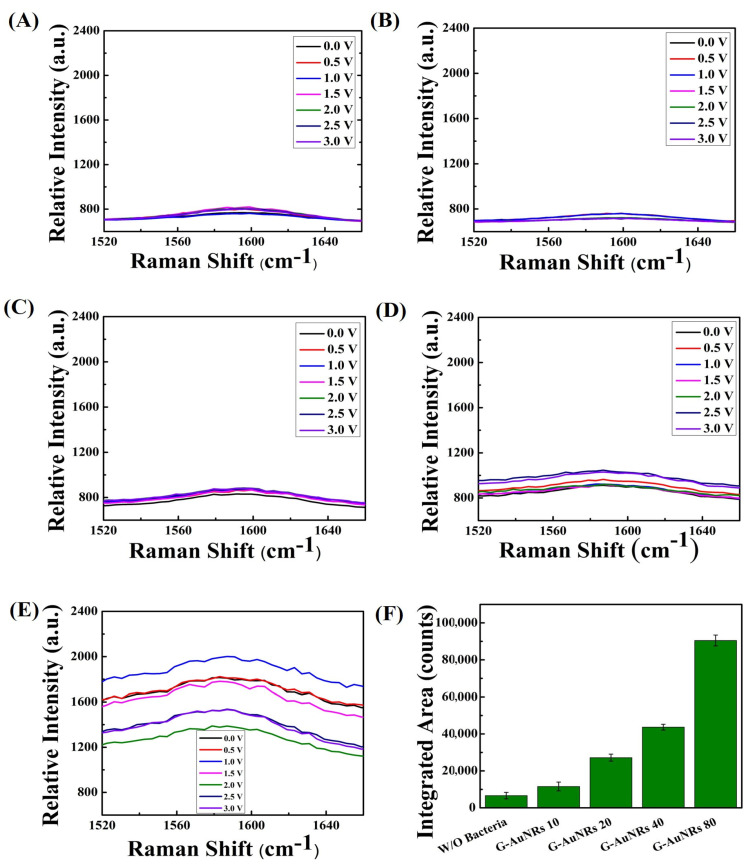
(**A**) The G band of graphene in the absence of bacteria (DI water) exhibited G band peaks at 1585 cm^−1^ with the concentration of AuNRs on graphene 80 µg/mL, 20 µL, 10 × 10 mm. (**B**–**E**) The G band of crumpled 3D graphene-AuNRs subjected to voltage modulation at varying concentrations (10, 20, 40, and 80 µg/mL), with a voltage range of 0 to 3 V applied. (**F**) The integrated area under the peak values, indicating distinct densities of AuNRs deposited on crumpled 3D graphene, for the G band of graphene interacting with Gram-negative *S. typhi* bacteria, utilizing a 3 V applied voltage.

However, the G band of graphene (~1585 cm^−1^) also showed a similar enhancement because of the superimposing of amide II (1555–1565 cm^−1^) [53] bands with the G band of graphene for both bacteria. No considerable enhancement was found when treated without bacterial suspension using DI water, (AuNRs density 80 µg/mL, 20 µL into 10 × 10 mm graphene) shown in Figure 4A and Figure 5A. The increasing AuNRs density on the surface of 3D graphene leads to an increment in the intensity of the D band for both bacteria, shown in Figure 4B–E and Figure 5B–E, respectively. The integrated area for SERS enhancement has been shown in Figure 4F and Figure 5F for the *Listeria monocytogenes* and *S. typhi*, respectively, which is drastically increased with the high density of AuNRs.

Gram-negative bacteria have an isoelectric point of approximately ~5 and Gram-positive bacteria of approximately ~3 [54]. So, it reflected the different electron states for the Gram-positive (Figure 2 and Figure 4) and the Gram-negative (Figure 3 and Figure 5) bacteria in the vibrational properties of graphene D and G bands. The highest intensity of graphene for Gram-negative bacteria for the D band and G band raises ~1280 a.u. and ~2010 a.u., respectively. In contrast, the highest intensity for the Gram-positive bacteria for the D and G bands of graphene are approximately ~1220 a.u. and ~1800 a.u. We plotted the large-area SERS enhancement for the D and G bands of both bacteria and found that the Gram-negative bacteria provides the large-area SERS enhancement compared to the Gram-positive bacteria because of the bacterial surface charge, shown in Figure 2F and Figure 4F for D, G band of graphene for the Gram-positive bacteria and Figure 3F and Figure 5F for the D, G band of graphene for the Gram-negative bacteria. The highest enhancement factor for Gram-negative bacteria is ~2.5 for the G band and ~2.1 for the D band when compared with and without bacteria whereas the enhancement factor for Gram-negative bacteria is ~1.6 and ~1.5 for the G and D band, respectively (Figure S7). The transmittance characteristics of the nanostructures showed a noticeable difference at 532 nm and 405 nm due to different resonant mechanisms controlling their optical nonlinearity. This leads to the influence of enhanced optical effects based on the carbon-based system with Au that can be further studied. The transmittance characteristics of the nanostructures showed a noticeable difference at 532 nm and 405 nm due to different resonant mechanisms controlling their optical nonlinearity [55]. This leads to the influence of enhanced optical effects based on the carbon-based system with Au that can be further studied.

### 3.3. Differentiation of Bacteria and Concentration-Based Study

**Figure 6:**
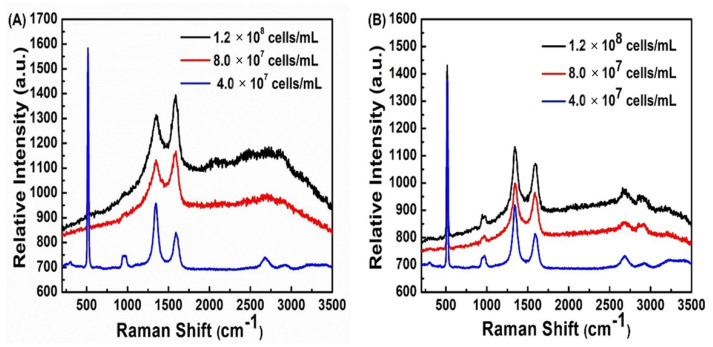
Raman spectra of graphene with bacterial samples of (**A**) *S. typhi* and (**B**) *Listeria monocytogenes* on different concentrations of bacterial suspension on 3 V with the density of AuNRs on the graphene 80 µg/mL, 20 µL, 10 × 10 mm and the three different concentrations of bacteria are 1.2 × 10^8^, 8.0 × 10^7^, 4.0 × 10^7^ cells/mL.

To closely figure out the concentration effects of the bacteria on the SERS platform, we performed concentration-based SERS analysis for both bacteria. The result shows the close binding of the Gram-negative to the AuNRs and graphene by charge transfer and the increasing concentration of the bacteria provides higher Raman intensity whereas the Gram-positive bacteria showed less affinity compared to the Gram-negative bacteria (Figure 6). The differences in the chemical composition of the bacterial cell wall for *Listeria monocytogenes* and *S. typhi* bacteria and their interaction with the 3D graphene-AuNRs at different concentrations may affect the intensity ratio of D and G bands. A Gram-negative bacterial cell has a thin peptidoglycan layer and a surface membrane made up of proteins, phospholipids, and lipopolysaccharides, whereas a Gram-positive cell has a thick peptidoglycan layer made up of teichoic and lipoteichoic acids. Gram-negative cells have an additional layer of the outer membrane while having a much thinner coating of peptidoglycan. In contrast to Gram-positive bacteria (*Listeria monocytogenes*), Gram-negative bacteria (*S. typhi*) maintain a stronger cell wall that offers higher resistance to the sharp edges of graphene. At higher concentrations of bacteria, the D band intensity is typically higher than the G band intensity, showing a more disordered graphene structure. This could be due to several factors, such as increased physical contact between the bacteria and the graphene surface [56] The D and G bands are characteristic peaks that correspond to the vibrational modes of carbon-based materials. D band arises because of the breathing modes of sp^2^ hybridized carbon atoms, while the G band corresponds to the stretching modes of the same. When bacteria are present on the graphene-based material, the interactions between the bacteria and the graphene surface can affect the vibrational modes of the graphene sheet. The intensity ratio of the D and G bands in Raman spectroscopy can provide information about disorders in the graphene structure [57]. The precise mechanisms underlying this phenomenon are complex and depend on a variety of factors, including the concentration of bacteria and the nature of the interaction between the bacteria and the graphene surface. However, the above demonstration of this experiment is based on the SERS platform integrated with a DC power supply by using AuNRs and graphene as SERS active materials to analyze the bacterial suspension at a very low amount and concentration, the Raman spectra of the bacterial suspension was got by nanometric thickness on of the bacterial cell wall that differentiates the type of bacteria. So, this method can still also apply to a single bacterium analysis by trapping the bacterium inside a microfluidic channel and analyzing its Raman spectra, etc. Our method can effectively detect low concentrations of bacterial suspension using graphene and AuNR. This is helpful for label-free biosensing compared to existing SERS-based methods [58]. However, we have compared the Raman spectra of 3D graphene-AuNRs (80 µg/mL) without and with mixed a bacterial suspension shown in Figure S8 as a perspective to analyze real samples and determine the percentage count of different bacteria in a sample.

## 4. Conclusions

In summary, we have shown a voltage-gated liquid Raman setup and analyzed the effect of AuNRs density on flake-like 3D graphene for label-free bacteria sensing at low concentrations of bacteria. The deposition of the AuNRs on the graphene enables the large-area SERS enhancement while handling the liquid samples. Our results showed the relationship between the AuNRs density on the graphene and the rate of integrated area enhancement of SERS and the SERS enhancement factor when comparing with and without bacterial suspension with a concentration as low as 4.0 × 10^7^ cells/mL. This demonstration showed that for a 80 µg/mL conc. of AuNR sample and 20 µL, the deposition volume on 10 × 10 mm of graphene surface provides the highest SERS integrated area enhancement. Additionally, the bacterial concentration-based study will provide significant information for the further development of label-free bacterial biosensing devices for quantitative studies. In our current system, we have not used polarization-controlled light sources for Raman measurement. We could perform further studies using a polarization-controlled light system to develop a novel Raman biosensor system.

Our work could clear the way for the application of Raman spectroscopy for the rapid analysis of the bacterial samples for clinical samples as we showed the liquid bacterial sample analysis as a label-free biosensor—for example, the rapid and quantitative bacterial sample analysis directly after collecting the sample from the patients. Additionally, the liquid-SERS enables a culture-free and less time-consuming method to analyze the liquid samples and provides a foundation for label-free and quantitative bacterial sample analysis.

## Data Availability

The authors confirm that the data supporting the findings of this study are available within the article and the raw data that support the findings are available from the corresponding author, upon reasonable request.

## Supplementary Materials

The following supporting information can be downloaded at: https://www.mdpi.com/article/10.3390/bios13110962/s1, Figure S1: (A) UV–vis spectra of AuNRs; (B) FTIR spectra of hydrophobic AuNRs, showing the thiol functional group at 2550–2600 cm^−1^; (C) visual observations (I) before (II) after the hydrophobication process of AuNRs; (D) redispersion of hydrophobic AuNRs into an organic solvent. Figure S2. (A) Standard Raman spectrum of synthesized flake-like 3D graphene showing the spectra of D (~1370 cm^−1^), G (~1585 cm^−1^), and 2D (~2700 cm^−1^) bands of graphene; (B) SEM image of directly grown flake-like 3D graphene with rough edges; (C,D) TEM images of AuNRs showing the rod-like structure. Figure S3. Atomic force microscopic images of (A) 3D graphene without deposition of AuNRs. Deposited AuNRs of different densities: (B) 10 µg/mL, (C) 20 µg/mL, (D) 40 µg/mL, and (E) 80 µg/mL on the graphene surface with the surface area of the device 10 × 10 mm and the distance analyzed by AFM is 5 µm. Figure S4. Raman spectra of crumpled 3D graphene-AuNRs (160 µg/mL) for the Gram-positive *Listeria monocytogenes:* (A) D band (~1370 cm^−1^) and (B) G band (~1585 cm^−1^) of graphene. Figure S5. Raman spectra of crumpled 3D graphene-AuNRs (160 µg/mL) for the Gram-negative *S. typhi:* (A) D band (~1370 cm^−1^) and (B) G band (~1585 cm^−1^) of graphene. Figure S6. Comparison of the Area under the peak values of (A) D band ~1370 cm^−1^ and (B) G band ~1585 cm^−1^ for Gram-positive *Listeria monocytogenes,* and (C) D band ~1370 cm^−1^ and (D) G band ~1585 cm^−1^ for Gram-negative *S. typhi* bacteria when different densities of AuNRs (10, 20, 40, 80, and 160 µg/mL) are deposited on a crumpled 3D graphene surface when a voltage of 3 V is applied. Figure S7. Enhancement factor (I/I0), where I denotes the Raman signal intensity with bacteria and I_0_ is without bacteria. Enhancement factor for D and G band of graphene with (A) Gram-positive *Listeria monocytogenes* and (B) Gram-negative *S. typhi*. Figure S8. Comparison with the Raman spectra of 3D graphene-AuNRs (80 µg/mL) without bacteria (A) and a mixed bacteria suspension of Gram-positive *Listeria monocytogenes* and Gram-negative *S. typhi* using 3D graphene-AuNRs (80 µg/mL) (B); the voltage ranges from 0 to 3 V.
