# Supplementary material for: Gold Nanorod Density-Dependent Label-Free Bacteria Sensing on a Flake-like 3D Graphene-Based Device by SERS
Source: Biosensors (Basel). 2023 Oct 30;13(11):962. doi: 10.3390/bios13110962 (PMC10669247; doi:10.3390/bios13110962)
Supplement: Supplementary file 1 [file biosensors-13-00962-s001.zip › biosensors-2642926-supplementary.pdf]

Supplementary Material

Gold Nanorod Density-Dependent Label-Free Bacteria Sensing on a Flake-Like 3D Graphene-Based Device by SERS

Md Imran Hossain, Sitansu Sekhar Nanda, Sooheon Cho, Bom Lee, Bum Jun Kim, Jae-Young Choi,* and Dong Kee Yi*

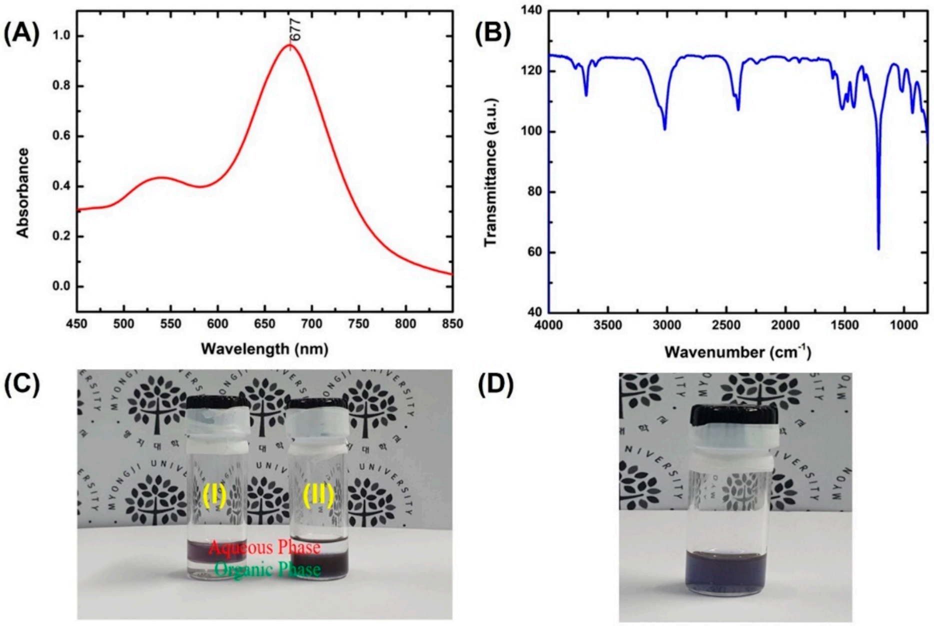

Figure S1. (A) UV–vis spectra of AuNRs; (B) FTIR spectra of hydrophobic AuNRs, showing the thiol functional group at 2550–2600 cm^{-1}; (C) visual observations (I) before (II) after the hydrophobication process of AuNRs; (D) redispersion of hydrophobic AuNRs into an organic solvent.

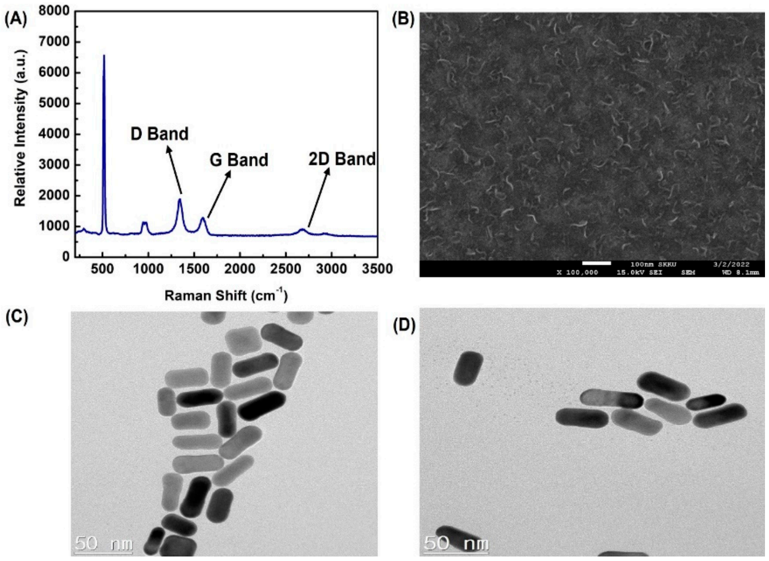

Figure S2. (A) Standard Raman spectrum of synthesized flake-like 3D graphene showing the spectra of D (~1370 cm^{-1}), G (~1585 cm^{-1}), and 2D (~2700 cm^{-1}) bands of graphene; (B) SEM image of directly grown flake-like 3D graphene with rough edges; (C, D) TEM images of AuNRs showing the rod-like structure.

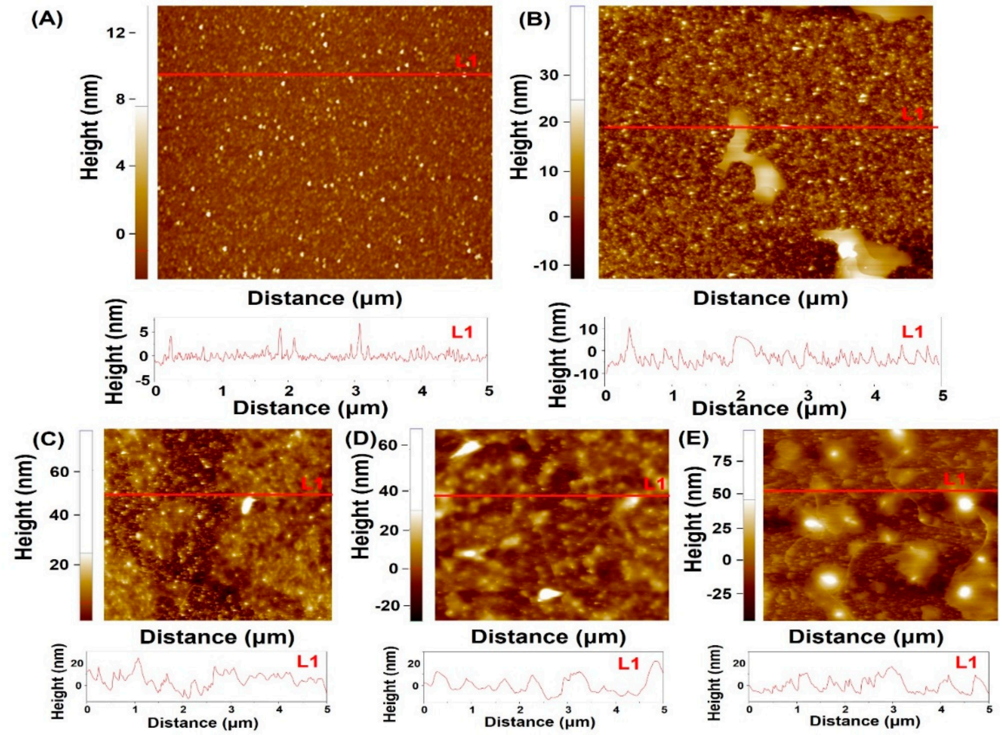

Figure S3. Atomic force microscopic images of (A) 3D graphene without deposition of AuNRs. Deposited AuNRs of different densities: (B) 10 μg/m,L (C) 20 μg/mL, (D) 40 μg/mL, and (E) 80 μg/mL on the graphene surface with the surface area of the device 10 X 10 mm and the distance analyzed by AFM is 5 μm.

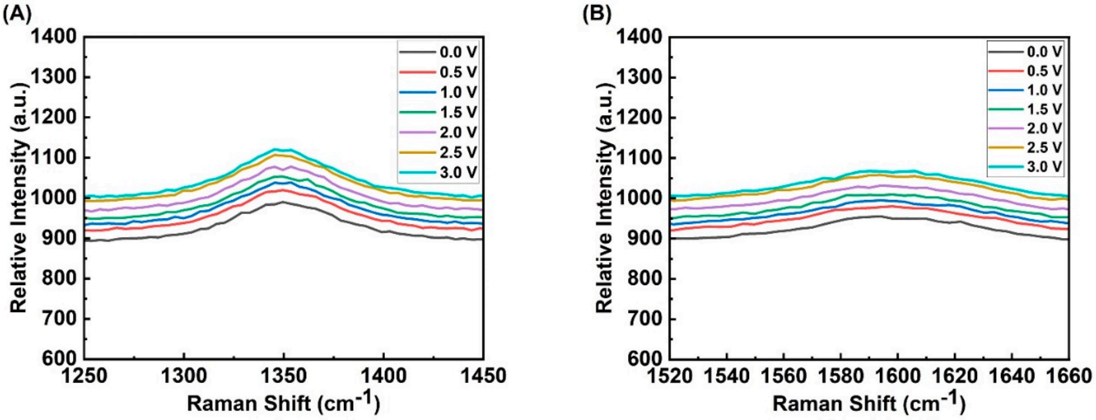

Figure S4. Raman spectra of crumpled 3D graphene-AuNRs (160 μg/mL) for the Gram-positive Listeria monocytogenes: (A) D band (~1370 cm^{-1}) and (B) G band (~1585 cm^{-1}) of graphene.

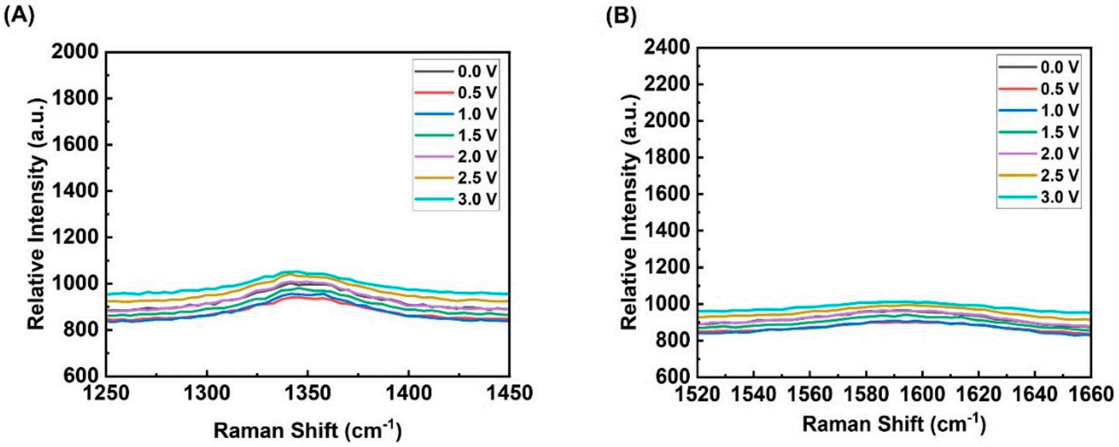

Figure S5. Raman spectra of crumpled 3D graphene-AuNRs (160 µg/mL) for the Gram-negative *S. typhi* A): D band (~1370 cm^{-1}) and (B) G band (~1585 cm^{-1}) of graphene.

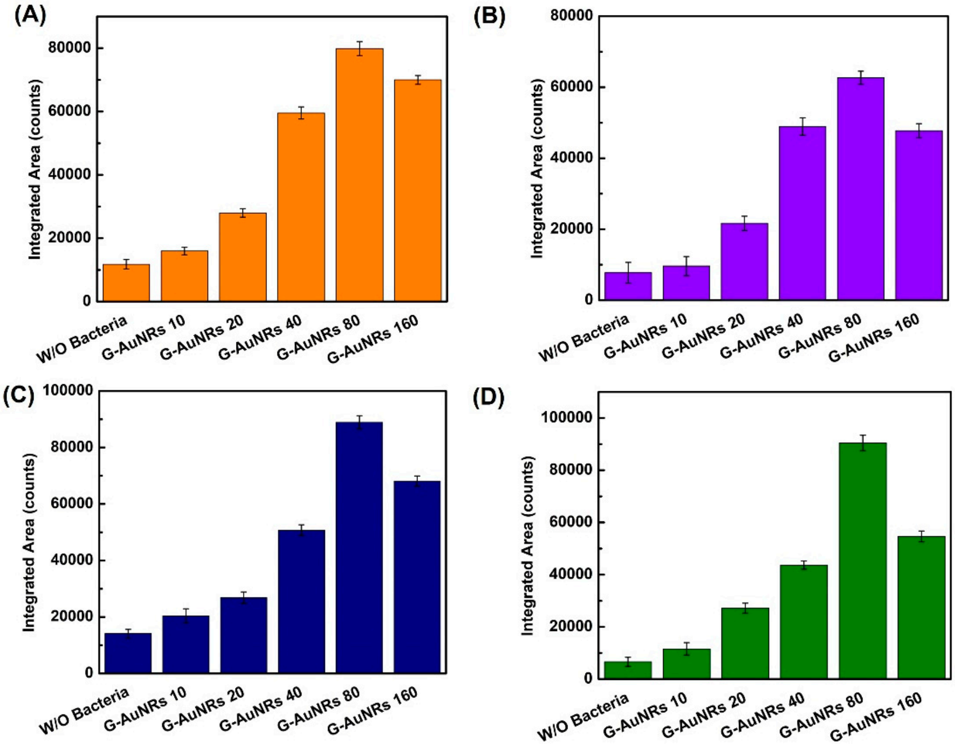

Figure S6. Comparison of the area under the peak values of (A) D band ~1370 cm^{-1} and (B) G band ~1585 cm^{-1} for Gram-positive *Listeria monocytogenes*, and (C) D band ~1370 cm^{-1} and (D) G band ~1585 cm^{-1} for Gram-negative *S. typhi* bacteria when different densities of AuNRs (10, 20, 40, 80, 160 µg/mL) are deposited on crumpled 3D graphene surface when a voltage applied of 3 V.

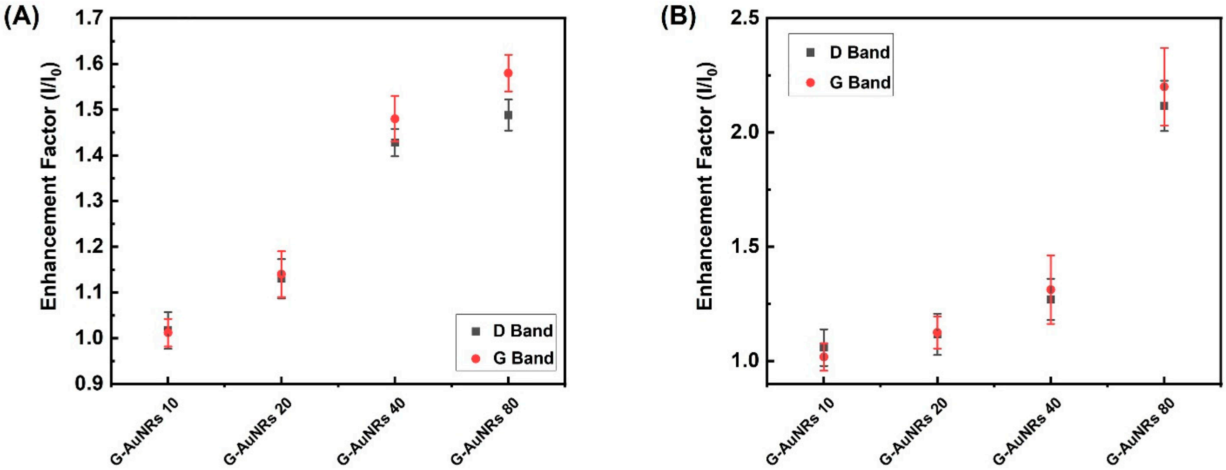

Figure S7. Enhancement factor (I/I_0), where *I* denotes the Raman signal intensity with bacteria and I_0 is without bacteria. Enhancement factor for D and G band of graphene with (A) Gram-positive *Listeria monocytogenes* and (B) Gram-negative *S. typhi*.

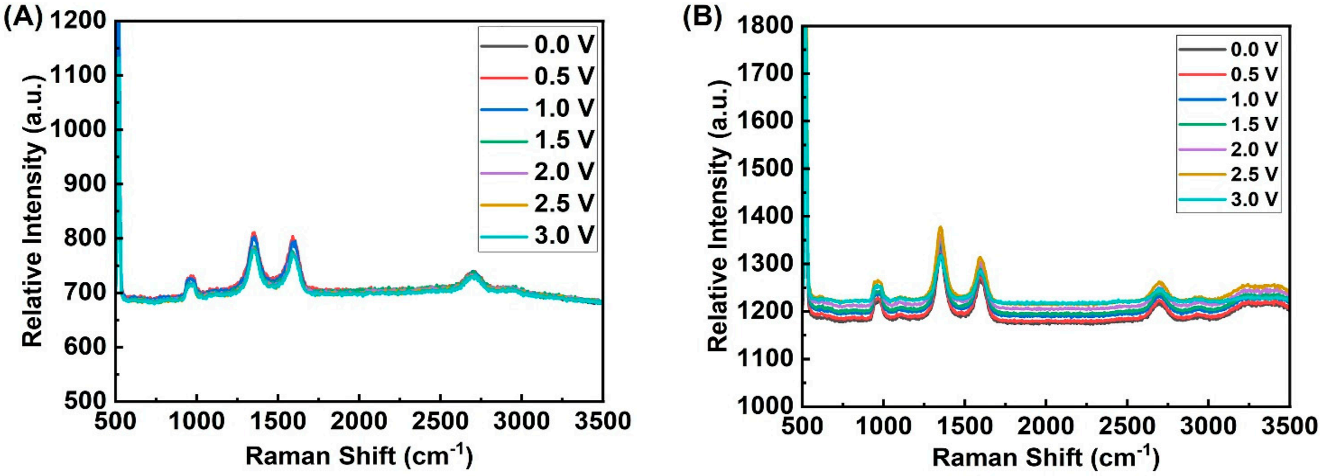

Figure S8. Comparison with the Raman spectra of 3D graphene-AuNRs (80 μg/mL) without bacteria (A) and a mixed bacteria suspension of Gram-positive *Listeria monocytogenes* and Gram-negative *S. typhi* using 3D graphene-AuNRs (80 μg/mL) (B); voltage ranges from 0 to 3 V.
